# Development of a benchmarking dataset for symptom detection using large language models

**DOI:** 10.1093/jamiaopen/ooag134

**Published:** 2026-07-10

**Authors:** Joshua Davis, Brigitte N Durieux, Chloe Van Dongen, Kate Sciacca, Juan Manuel Gutierrez, Charlotta Lindvall

**Affiliations:** Department of Supportive Oncology, Dana-Farber Cancer Institute, Boston, MA, 02215, United States; Albany Medical College, Albany, NY, 12208, United States; Department of Supportive Oncology, Dana-Farber Cancer Institute, Boston, MA, 02215, United States; Department of Supportive Oncology, Dana-Farber Cancer Institute, Boston, MA, 02215, United States; Department of Supportive Oncology, Dana-Farber Cancer Institute, Boston, MA, 02215, United States; Department of Informatics & Analytics, Dana-Farber Cancer Institute, Boston, MA, 02215, United States; Department of Supportive Oncology, Dana-Farber Cancer Institute, Boston, MA, 02215, United States; Harvard Medical School, Harvard University, Boston, MA, 02115, United States

**Keywords:** natural language processing, artificial intelligence, computing methodologies, signs and symptoms, symptom assessment

## Abstract

**Objectives:**

To develop a pipeline for evaluating large language models (LLMs) on the task of capturing symptoms from clinical encounters.

**Materials and Methods:**

We created a gold standard dataset of symptom annotations from simulated doctor-patient encounter excerpts (264 encounters; 16 symptoms; double-coded and adjudicated). Nine different LLMs from 4 vendors (OpenAI, Meta, DeepSeek, Moonshot AI) were used as examples to test our evaluation pipeline; outputs were assessed for correct structure and symptom information.

**Results:**

Of 3085 excerpts, 2087 (68%) contained symptoms. Pain, cough, and shortness of breath were most common; LLMs achieved F1 scores ranging 0.66-0.88 for these symptoms with minimal prompt engineering. Of tested models, GPT-4.1 demonstrated the best overall performance.

**Discussion:**

Our evaluation pipeline and benchmarking dataset are publicly available and applicable to various LLMs, including open-source models.

**Conclusion:**

This work supports the development and optimization of models that seek to improve patient symptom understanding.

## Background and significance

Patient-reported outcome surveys are commonly used to bridge the gap between patient experience and clinical understanding, especially in regard to patients’ symptoms.^1^^,^^2^ However, their implementation often faces challenges such as survey fatigue, lack of engagement, and unclear purpose, resulting in variable response rates.^3^ These limitations highlight the need for innovative approaches to enhance patient-centered symptom tracking: 1 potential solution involves leveraging audio recording, transcription, and automated extraction to supplement traditional data collection methods and improve data quality.

Benchmarking, a standard practice in the development of large language models (LLMs), involves comparing a model’s outputs with “ground truth” data to quantify performance.^4^ This evaluation process supports performance monitoring, error detection, and model selection, enabling consistent comparisons and informed decisions, such as linking model or prompt changes to task improvements. While comprehensive evaluation of LLMs for clinical applications necessitates human review and qualitative analysis,^5^ quantitative benchmarking methods play a crucial role in the initial stages of model development and optimization.

## Objective

Our primary goal was to develop a reusable methodology and benchmarking dataset for evaluating LLM performance on clinical symptom detection tasks. We also demonstrate its utility through a proof-of-concept analysis with 9 models from 4 vendors.

## Methods

### Overview

**Figure 1.**
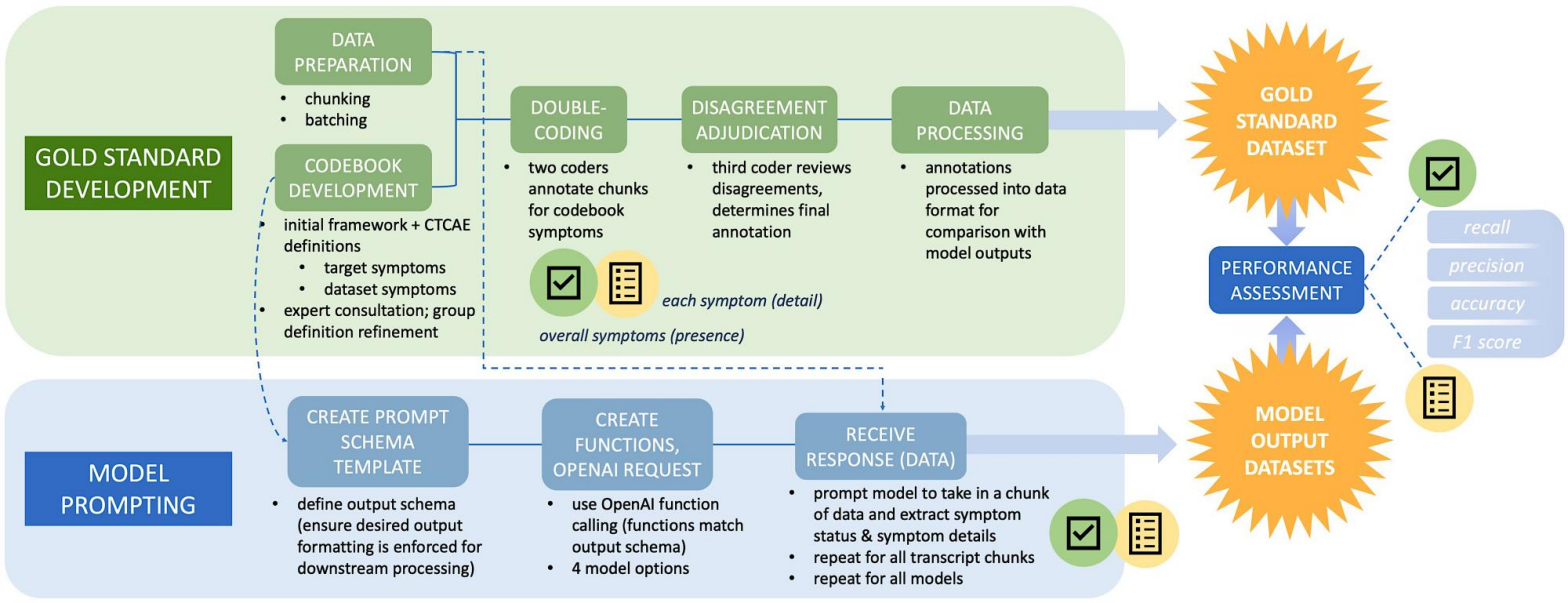
Overview of methods. A visual overview of the process followed to create the benchmarking dataset and associated processing pipeline described in this paper.

This is a secondary proof-of-concept analysis of 264 publicly available synthetic conversations.^6^ First, we created a gold standard benchmarking dataset for 16 symptoms of interest at the excerpt level (Figure 1): we report interrater reliability and provide our final dataset. Second, we developed a programmatic pipeline for LLM data extraction via application programming interface (API), enabling use of the benchmarking dataset for performance assessment and model comparisons: we demonstrate this pipeline with 9 LLMs, reporting performance information and observations.

### Data

This study used publicly available synthetic conversation transcripts from Fareez et al.^6^ comprising 272 simulated patient-physician interviews in objective structured clinical examination format. Eight conversations were excluded from the gold standard annotation process due to encoding issues found during quality review (*n* = 2) or use in initial data familiarization and codebook development steps (*n* = 6). To minimize privacy concerns associated with LLM usage,^7^ we used simulated conversation data. As a result of this, demographic information was not associated with any of the participants.

### Preprocessing

Large language models generate outputs based on specific instructions largely given in a prompt (ie, input) to the model. Preliminary work with LLMs emphasized the importance of limiting the number of tokens associated with any prompt (input size). As LLMs tend to perform better regarding information seen at the beginning and end of input data,^8^ we split transcripts into nonoverlapping (<200 token) excerpts using RecursiveCharacterTextSplitter from langchain^9^ to minimize potential information loss.

### Symptom codebook and definitions

Our symptom annotations took place at 2 levels per excerpt: general (whether any symptoms were mentioned) and granular (whether specific symptoms were mentioned). Our initial codebook comprised 12 symptoms reported by patient-reported outcomes at Dana-Farber Cancer Institute (anxiety, constipation, diarrhea, fatigue, nausea, numbness and tingling, pain, poor appetite, rash, shortness of breath, trouble drinking fluids, and vomiting); symptom definitions arose from Common Terminology Criteria for Adverse Events definitions.^10^ This codebook was applied to excerpts from 6 conversations used for data familiarization; observations from this and subsequent tests were discussed with the larger study team, informing iterative refinements. These included the addition of 4 symptoms (headache, concentration problems, cough, fever) plus an “Other” category. Our final codebook comprised 16 specific symptoms (Table S1): this was applied by annotators to 264 conversations to create the gold standard dataset.

### Annotation process

To mitigate coder fatigue during the annotation process, excerpts were prepared and reviewed in small sets (batches). Three coders (B.N.D., C.V.D., K.S.) were involved in the annotation of each excerpt. Each excerpt was annotated separately by 2 of the coders using Label Studio,^11^ with results exported and disagreements adjudicated by the third coder using Excel.^5^ Questions and uncertain cases were discussed with the larger study team to reach consensus on the applied annotation. We assessed interrater reliability using intraclass correlation coefficient (ICC)^12^ for general symptom annotations and Cohen’s kappa^13^ for specific symptom annotations.

### Models

To demonstrate our pipeline, we tested 9 LLMs representing a variety of popular open- and closed-source models: including GPT-4.1 (OpenAI), GPT-4.1 Mini (OpenAI), GPT-4.1 Nano (OpenAI), GPT-4o Mini (OpenAI), DeepSeek-V3 (DeepSeek), Llama-3.1 8B (Meta), Llama-3.2 3B (Meta), Llama-3.3 70B (Meta), and Kimi K2 (Moonshot AI). We selected these models given their widespread popularity regarding exploring clinical LLM utility and accessibility;^14^ however, the gold standard dataset produced by this work enables benchmarking of other models and prompts in the future.

### Prompting

We drafted 2 zero-shot prompts for (1) detecting symptoms and (2) identifying specific symptoms (Table S2). The same prompts were used across all LLMs to rapidly assess for differences in performance between models. As the focus of this work was creating a dataset and replicable process for model evaluation, we did not engage in prompt optimization beyond necessary for preliminary benchmarking.

### Structured output

Enforcing a single output format enabled rapid comparison against our benchmarking dataset. We used the response format parameter for models served by both OpenAI and Together AI using the OpenAI API.^15^ This was done by using a pydantic schema as a parameter (for OpenAI, “text format,” for TogetherAI, “response format”) and subsequently validating the output using the same pydantic schema;^16^ a temperature of 0.0 was used to generate more consistent outputs.^17^ Outputs not matching the defined structure were set to raise an error, with the LLM retrying up to 5 times. Correctly formatted outputs were used in downstream analysis.

### Analyses

We calculated standard evaluation metrics (precision, recall, accuracy, F1 score) with 95% CIs using bootstrapping (1000 samples). To test for statistically significant differences in model performance, we used Cochran’s *Q* test (*α* = .05)^18^ followed by a post hoc pairwise McNemar’s test with Bonferroni correction for multiple comparisons (adjusted *α* ≈ .0014).^19^ For symptom-level analysis, we calculated false positive and false negative rates. To contextualize and gain insights into the nature of errors, we manually reviewed a random sample of GPT-4.1 errors encountered for 3 most common symptoms (15 false positives and 15 false negatives per symptom).

## Results

### Dataset

**Figure 2.**
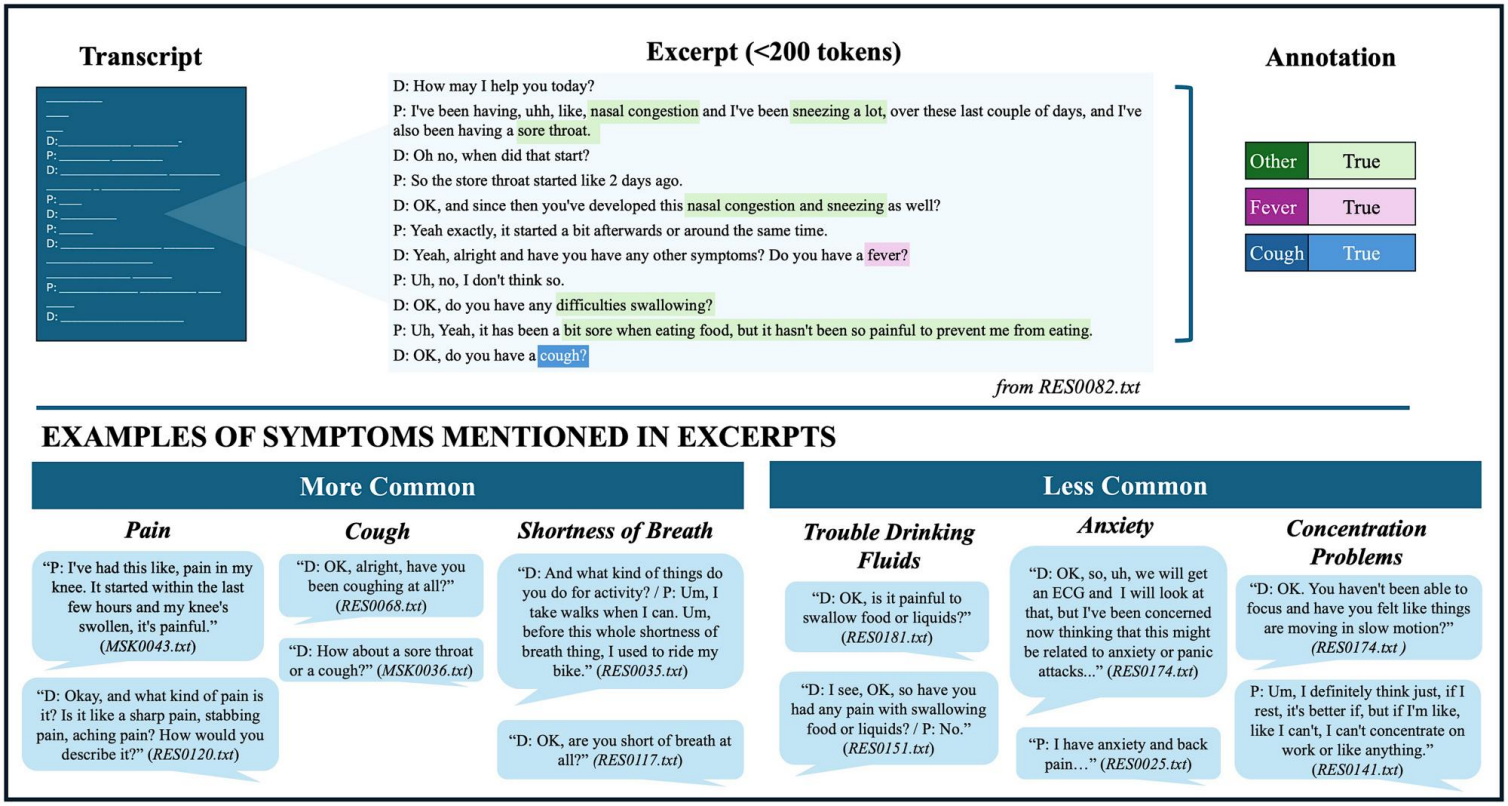
Examples of symptom mentions within transcript. This figure illustrates our annotation of excerpts and depicts examples of common and uncommon symptoms as they appear in the dataset. Note that examples for all symptoms are available in our codebook (Table S1).

Of 3085 excerpts, 2087 contained symptoms (Table 1). Human coders annotated for symptom presence/absence with high reliability, with an average ICC of 0.92. For specific symptom annotation, average agreement between pairs of coders was near perfect^13^ (Cohen’s *k = *0.91). The 3 most prevalent symptoms were pain (*n* = 983 excerpts, *k = *0.89), cough (*n* = 707, *k = *0.93), and shortness of breath (*n* = 520, *k = *0.95); example excerpts are shown in Figure 2.

**Table 1.** Data description.

Dataset		
Number of transcripts	264	
Number of excerpts	3085	
Average excerpts per transcript (*n*)	12	
Overall symptoms (presence/absence)		
Number of excerpts with symptoms (%)	2087 (68)	
Average ICC	0.92	

**Specific symptoms**	***n* (excerpts with symptoms appear in)**	***k* (average interrater reliability between coder pairs)**

Anxiety	15	0.71
Concentration problems	6	0.56
Constipation	116	0.98
Cough	707	0.93
Diarrhea	182	0.96
Fatigue	215	0.85
Fever	437	0.92
Headache	248	0.97
Nausea	219	0.99
Numbness and tingling	93	0.96
Pain	983	0.89
Poor appetite	138	0.68
Rash	198	0.95
Shortness of breath	520	0.95
Trouble drinking fluids	27	0.55
Vomiting	225	0.98
Other	1527	0.81

Excerpts were nonoverlapping (<200 token) sections of transcript text, annotated for the mention of the above symptoms. The “Other” category was used by annotators as a catch-all for mention of additional symptoms.

Abbreviations: ICC, intraclass correlation coefficient; *k*, Cohen’s kappa.

### Model performance

Cochrane’s *Q* test revealed significant performance differences across tested models (*Q* = 2.5e3, *P* < .05); post hoc McNemar tests compared the performance of each model vs the others (Table S3). Model performance on symptom detection and for the 3 most common symptoms is shown in Table 2. For detecting general mention of symptoms, GPT-4.1 performed highest of the 9 evaluated models, with a precision of 0.90, recall of 0.86, accuracy of 0.84, and F1 score of 0.88; for extracting mention of specific symptoms, performance varied across models and symptoms (Table 2, Table S4). For the 3 most common symptoms seen in the dataset, F1 scores ranged from 0.66 to 0.88 across models. Rarer symptoms (eg, trouble drinking fluids, concentration problems) typically saw reduced performance; small samples limited our ability to evaluate on these symptoms.

**Table 2.** Model performance.

		Precision (95% CI)	Recall (95% CI)	Accuracy (95% CI)	F1 score (95% CI)
Symptoms					
GPT-4.1 Mini (OpenAI)		0.85 (0.84-0.87)	0.89 (0.88-0.91)	0.82 (0.81-0.84)	0.87 (0.86-0.88)
GPT-4.1 (OpenAI)		**0.90 (0.89-0.91)**	0.86 (0.85-0.88)	0.84 (0.83-0.86)	0.88 (0.87-0.89)
GPT-4.1 Nano (OpenAI)		0.86 (0.84-0.87)	0.73 (0.71-0.75)	0.73 (0.72-0.75)	0.79 (0.77-0.80)
GPT-4o Mini (OpenAI)		0.84 (0.83-0.86)	0.84 (0.82-0.85)	0.78 (0.77-0.80)	0.84 (0.83-0.85)
Llama-3.1 (Meta)		0.69 (0.67-0.70)	**1.00 (1.00-1.00)**	0.69 (0.68-0.71)	0.82 (0.80-0.83)
Llama-3.2 (Meta)		0.67 (0.65-0.70)	0.42 (0.40-0.44)	0.47 (0.45-0.49)	0.52 (0.50-0.54)
Llama-3.3 (Meta)		0.78 (0.77-0.79)	**0.99 (0.99-1.00)**	0.81 (0.79-0.82)	0.87 (0.86-0.88)
DeepSeek-V3 (DeepSeek)		0.83 (0.82-0.85)	0.86 (0.85-0.88)	0.79 (0.78-0.81)	0.85 (0.84-0.86)
Kimi K2 (Moonshot AI)		0.82 (0.80-0.84)	0.89 (0.88-0.91)	0.79 (0.78-0.81)	0.85 (0.84-0.87)
Specific symptoms					
GPT-4.1 Mini	Pain	**0.91 (0.89-0.93)**	0.63 (0.59-0.65)	0.79 (0.78-0.81)	0.74 (0.72-0.76)
GPT-4.1		**0.90 (0.88-0.93)**	0.62 (0.59-0.65)	0.79 (0.77-0.81)	0.74 (0.71-0.76)
GPT-4.1 Nano		0.83 (0.81-0.86)	0.67 (0.64-0.70)	0.78 (0.76-0.80)	0.74 (0.72-0.77)
GPT-4o Mini		**0.91 (0.89-0.93)**	0.62 (0.59-0.65)	0.79 (0.77-0.81)	0.74 (0.71-0.76)
Llama-3.1		**0.92 (0.90-0.94)**	0.61 (0.58-0.64)	0.79 (0.78-0.81)	0.73 (0.71-0.76)
Llama-3.2		**0.94 (0.92-0.96)**	0.53 (0.50-0.56)	0.76 (0.75-0.78)	0.68 (0.65-0.71)
Llama-3.3		**0.91 (0.88-0.93)**	0.64 (0.61-0.67)	0.80 (0.78-0.82)	0.75 (0.72-0.77)
DeepSeek-V3		0.75 (0.72-0.78)	0.60 (0.56-0.63)	0.71 (0.69-0.73)	0.66 (0.64-0.69)
Kimi K2		0.84 (0.82-0.87)	0.69 (0.66-0.72)	0.79 (0.78-0.81)	0.76 (0.74-0.78)
GPT-4.1 Mini	Cough	**0.94 (0.92-0.96)**	0.82 (0.79-0.85)	0.92 (0.91-0.93)	0.87 (0.85-0.89)
GPT-4.1		**0.98 (0.97-0.99)**	0.80 (0.77-0.83)	0.93 (0.92-0.94)	0.88 (0.86-0.90)
GPT-4.1 Nano		0.84 (0.81-0.86)	0.86 (0.83-0.89)	0.90 (0.88-0.91)	0.85 (0.83-0.87)
GPT-4o Mini		**0.97 (0.96-0.98)**	0.81 (0.78-0.84)	0.93 (0.92-0.94)	0.88 (0.87-0.90)
Llama-3.1		**0.98 (0.97-0.99)**	0.80 (0.77-0.83)	0.93 (0.91-0.94)	0.88 (0.86-0.90)
Llama-3.2		**0.95 (0.94-0.97)**	0.81 (0.78-0.84)	0.92 (0.91-0.93)	0.87 (0.85-0.89)
Llama-3.3		**0.97 (0.95-0.98)**	0.81 (0.78-0.84)	0.93 (0.92-0.94)	0.88 (0.86-0.90)
DeepSeek-V3		**0.92 (0.90-0.94)**	0.78 (0.75-0.81)	0.90 (0.89-0.92)	0.84 (0.82-0.87)
Kimi K2		0.89 (0.86-0.91)	0.84 (0.81-0.86)	0.91 (0.90-0.92)	0.86 (0.84-0.88)
GPT-4.1 Mini	Shortness of breath	0.92 (0.89-0.95)	0.65 (0.61-0.70)	0.90 (0.89-0.91)	0.76 (0.73-0.80)
GPT-4.1		0.92 (0.89-0.95)	0.63 (0.59-0.67)	0.89 (0.88-0.91)	0.75 (0.72-0.78)
GPT-4.1 Nano		0.85 (0.82-0.89)	0.68 (0.64-0.72)	0.89 (0.88-0.90)	0.75 (0.73-0.78)
GPT-4o Mini		0.92 (0.89-0.95)	0.65 (0.60-0.69)	0.90 (0.89-0.91)	0.76 (0.73-0.79)
Llama-3.1		**0.98 (0.96-0.99)**	0.62 (0.57-0.66)	0.90 (0.89-0.91)	0.76 (0.72-0.79)
Llama-3.2		0.94 (0.92-0.97)	0.66 (0.62-0.70)	0.90 (0.89-0.92)	0.78 (0.74-0.81)
Llama-3.3		0.93 (0.91-0.96)	0.67 (0.63-0.71)	0.91 (0.89-0.92)	0.78 (0.75-0.81)
DeepSeek-V3		0.94 (0.91-0.96)	0.61 (0.57-0.65)	0.89 (0.88-0.91)	0.74 (0.71-0.77)
Kimi K2		0.85 (0.82-0.88)	0.68 (0.64-0.72)	0.89 (0.88-0.90)	0.76 (0.73-0.79)

Performance data for general symptoms and the 3 most common symptoms shown here. Performance data for all symptoms is available in Table S4. Values of 0.90 and above are bolded.

### Errors

**Figure 3.**
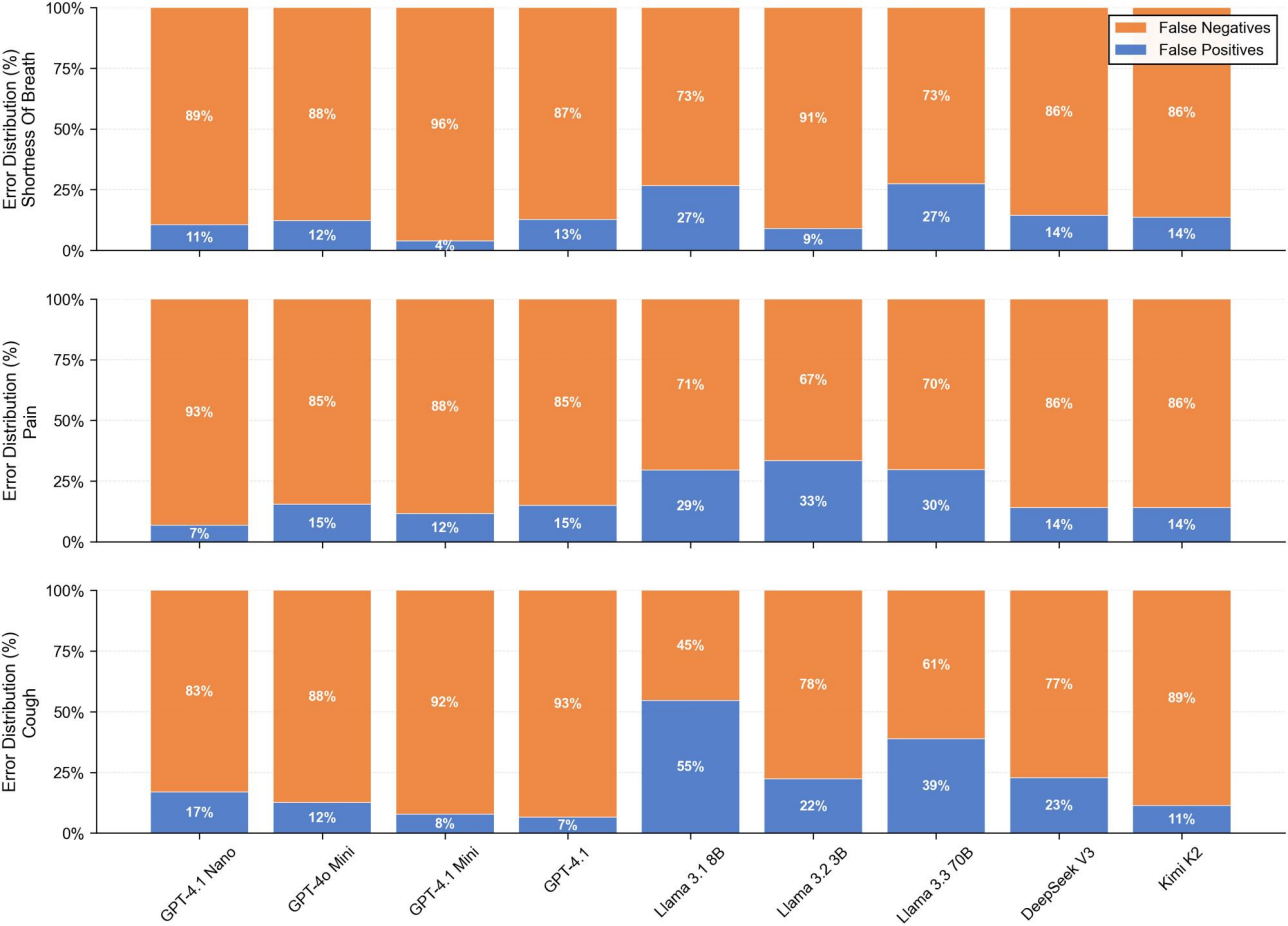
Relative distribution of error metrics across different models. This figure displays percentage distribution of error for all models’ detection of 3 symptoms most commonly observed in the dataset.

Across models for each of the top 3 symptoms, there was a higher rate of false negatives (failure to capture a symptom) to false positives (incorrectly detected symptom) (Figure 3, Table S5). The only exception involved Llama-3.1 8B (Meta) encountering more false positives than false negatives when identifying cough; manual error review suggests this to be largely due to the simplicity of the tested LLM prompt not accounting for decision-making guidance around ambiguous or edge cases (eg, mention of cold, coughing up blood, bringing up sputum, etc.).

### Structured output

All outputs were delivered by all models in the correct format.

## Discussion

In this proof-of-concept analysis of transcribed clinical conversations, we developed a benchmarking dataset and pipeline to evaluate the performance of LLMs on 2 symptom detection tasks. The pipeline facilitated a streamlined assessment of 9 different LLMs; for excerpt-level symptom detection, GPT-4.1 was the best-performing model, and for granular symptom extraction, performance varied across models. These initial results highlight the potential of leveraging optimized LLMs for symptom detection, as the current performance values reflect the testing of very basic prompts.

Although this study includes preliminary benchmarking result to showcase the dataset and pipeline’s utility, its primary aim is to provide accessible tools for the broader research community. These resources are intended to support future studies and enable comparative evaluations of LLMs.

To our knowledge, this study presents the largest set of publicly available annotated doctor-patient transcripts without protected health information, enabling LLM performance exploration around symptoms without privacy concerns. Other researchers have benchmarked similar tasks; for instance, Sushil et al. provide a public benchmarking dataset of oncologic information, including symptoms, from 40 anonymized clinical notes of patients with breast and pancreatic cancer.^20^ Building on Rhouma et al.’s use of the same source transcripts to evaluate BERT and FLAN-T5 models,^21^ we created our own dataset with detailed symptom annotations and a transparent benchmark pipeline to enhance reproducibility and support further research.

Our findings build on and extend prior research on automated symptom detection from clinical texts,^22–27^ including our previous work exploring both rule-based and deep learning methods to detect symptoms in patients with cancer.^22^^,^^23^ While these methods have demonstrated utility, they face limitations in generalizability and scalability due to their reliance on specific private datasets or rule-based systems. Deep learning models, too, may struggle with transferability across different clinical contexts. Large language models offer greater flexibility and broader language understanding; recent studies have highlighted their potential for extracting structured information from unstructured medical data.^28–31^

Future work will focus on adapting the pipeline for real-world clinical data to improve external validity, as well as optimizing LLM performance for symptom detection through fine-tuning and prompt engineering. Additionally, methods for aggregating excerpt-level scores into conversation-level annotations should be explored to provide a more comprehensive view of symptom discussions. Expanding the pipeline to support qualitative and task-based evaluations, including clinician feedback, will further ensure its clinical utility and alignment with real-world needs.

### Limitations

The transcripts used in this study represented simulated encounters which, while beneficial from a security standpoint, may lack external validity. The dataset was derived largely from published respiratory cases; more diverse clinical data would enhance generalizability in future iterations. Lacking demographic data, we were unable to investigate biases and performance discrepancies, a concern for LLMs and clinical natural language processing tools.^32–34^ This necessitates investigation of model performance on various real patient data. Additionally, preprocessing the data into 200-token chunks may have influenced LLM performance; future benchmarking should explore optimal context window sizes across models. Lastly, while this benchmarking dataset enables quantitative assessment of LLM performance, qualitative, mixed-methods, and task-based evaluations are needed to fully assess clinical utility and patient impact prior to implementation.^5^ These limitations are relevant to any LLM-based tool intended for clinical application. However, by offering high-quality symptom annotations accompanying simulated transcripts, this study provides a valuable foundation for preliminary evaluations prior to tests on real patient data.

## Conclusions

This work provides a publicly available benchmarking dataset and reproducible pipeline, enabling researchers to explore and optimize LLMs for clinical symptom detection. Future studies can use these resources to expand upon our findings and explore real-world applications.

## Supplementary Material

ooag134_Supplementary_Data

## Data Availability

The annotated dataset and supporting code are available on Github: https://github.com/lindvalllab/symptom-extraction-demo.

## References

[1] Basch E , Deal AM, Kris MG, et al Symptom monitoring with patient-reported outcomes during routine cancer treatment: a randomized controlled trial. J Clin Oncol. 2016;34:557-565. 10.1200/JCO.2015.63.0830 26644527 PMC4872028

[2] Lu DJ , Girgis M, David JM, et al Evaluation of mobile health applications to track patient-reported outcomes for oncology patients: a systematic review. Adv Radiat Oncol. 2021;6:100576. 10.1016/j.adro.2020.09.016 33073061 PMC7547022

[3] Sokas C , Hu F, Edelen M, et al A review of PROM implementation in surgical practice. Ann Surg. 2022;275:85-90. 10.1097/SLA.0000000000005029 34183512

[4] Thiyagalingam J , Shankar M, Fox G, et al Scientific machine learning benchmarks. Nat Rev Phys. 2022;4:413-420. 10.1038/s42254-022-00441-7

[5] Moreno AC , Bitterman DS. Toward clinical-grade evaluation of large language models. Int J Radiat Oncol Biol Phys. 2024;118:916-920. 10.1016/j.ijrobp.2023.11.012 38401979 PMC11221761

[6] Fareez F , Parikh T, Wavell C, et al A dataset of simulated patient-physician medical interviews with a focus on respiratory cases. Sci Data. 2022;9:313. 10.1038/s41597-022-01423-1 35710769 PMC9203765

[7] Umeton R , Kwok A, Maurya R, et al GPT-4 in a Cancer Center–Institute-wide deployment challenges and lessons learned. NEJM AI. 2024;1:AIcs2300191. 10.1056/AIcs2300191

[8] Liu NF , Lin K, Hewitt J, et al Lost in the middle: how language models use long contexts. arXiv, arXiv:2307.03172, 2023, preprint: not peer reviewed.

[9] Chase H. LangChain. 2022.

[10] U.S. Department of Health and Human Services, National Institutes of Health, National Cancer Institute. Common Terminology Criteria for Adverse Events (CTCAE), Version 4.0. 2009.

[11] Tkachenko M , Malyuk M, Shevchenko N, et al Label Studio: data labeling software. 2020.

[12] Bartko JJ. The intraclass correlation coefficient as a measure of reliability. Psychol Rep. 1966;19:3-11. 10.2466/pr0.1966.19.1.3 5942109

[13] Cohen J. A coefficient of agreement for nominal scales. Educ Psychol Meas. 1960;20:37-46. 10.1177/001316446002000104

[14] Rao A , Pang M, Kim J, et al Assessing the utility of ChatGPT throughout the entire clinical workflow. 2023. 10.2196/48659 PMC10481210 37606976

[15] OpenAI. OpenAI API. OpenAI Platf. Accessed July 15, 2024. https://platform.openai.com/docs/overview

[16] Pydantic. Pydantic Doc. Version V282. Accessed July 15, 2024. https://docs.pydantic.dev/latest/

[17] Davis J , Van Bulck L, Durieux BN, et al The temperature feature of ChatGPT: modifying creativity for clinical research. JMIR Hum Factors. 2024;11:e53559. 10.2196/53559 38457221 PMC10960206

[18] Cochran WG. The comparison of percentages in matched samples. Biometrika. 1950;37:256-266. 10.2307/2332378 14801052

[19] McNemar Q. Note on the sampling error of the difference between correlated proportions or percentages. Psychometrika. 1947;12:153-157. 10.1007/BF02295996 20254758

[20] Sushil M , Kennedy VE, Mandair D, et al CORAL: expert-curated medical oncology reports to advance language model inference. NEJM AI. 2024;1. 10.1056/AIdbp2300110 PMC12007910 40255242

[21] Rhouma R , McMahon C, Mcgillivray D, et al Leveraging mobile NER for real-time capture of symptoms, diagnoses, and treatments from clinical dialogues. Inform Med Unlocked. 2024;48:101519. 10.1016/j.imu.2024.101519

[22] Lindvall C , Deng C-Y, Agaronnik ND, et al Deep learning for cancer symptoms monitoring on the basis of electronic health record unstructured clinical notes. JCO Clin Cancer Inform. 2022;6:e2100136. 10.1200/CCI.21.00136 35714301 PMC9232368

[23] Durieux BN , Zverev SR, Tarbi EC, et al Development of a keyword library for capturing PRO-CTCAE-focused “symptom talk” in oncology conversations. JAMIA Open, 2023;6:ooad009. 10.1093/jamiaopen/ooad009 36789287 PMC9912707

[24] DiMartino L , Miano T, Wessell K, et al Identification of uncontrolled symptoms in cancer patients using natural language processing. J Pain Symptom Manage. 2022;63:610-617. 10.1016/j.jpainsymman.2021.10.014 34743011 PMC8930509

[25] Leiter RE , Santus E, Jin Z, et al Deep natural language processing to identify symptom documentation in clinical notes for patients with heart failure undergoing cardiac resynchronization therapy. J Pain Symptom Manage. 2020;60:948-958.e3. 10.1016/j.jpainsymman.2020.06.010 32585181

[26] Dreisbach C , Koleck TA, Bourne PE, et al A systematic review of natural language processing and text mining of symptoms from electronic patient-authored text data. Int J Med Inform. 2019;125:37-46. 10.1016/j.ijmedinf.2019.02.008 30914179 PMC6438188

[27] Nishiyama T , Yamaguchi A, Han P, et al Automated system to capture patient symptoms from multitype Japanese clinical texts: retrospective study. JMIR Med Inform. 2024;12:e58977. 10.2196/58977 39316418 PMC11462096

[28] Truhn D , Loeffler CM, Müller-Franzes G, et al Extracting structured information from unstructured histopathology reports using generative pre-trained transformer 4 (GPT-4). J Pathol. 2024;262:310-319. 10.1002/path.6232 38098169

[29] Adams LC , Truhn D, Busch F, et al Leveraging GPT-4 for post hoc transformation of free-text radiology reports into structured reporting: a multilingual feasibility study. Radiology. 2023;307:e230725. 10.1148/radiol.230725 37014240

[30] Zeinali N , Albashayreh A, Fan W, et al Symptom-BERT: enhancing cancer symptom detection in EHR clinical notes. J Pain Symptom Manage. 2024;68:190-198.e1. 10.1016/j.jpainsymman.2024.05.015 38789092 PMC12433187

[31] Patel PV , Davis C, Ralbovsky A, et al Large language models outperform traditional natural language processing methods in extracting patient-reported outcomes in inflammatory bowel disease. Gastro Hep Adv. 2024;4. 10.1016/j.gastha.2024.10.003 PMC11772946 39877865

[32] Zack T , Lehman E, Suzgun M, et al Assessing the potential of GPT-4 to perpetuate racial and gender biases in health care: a model evaluation study. Lancet Digit Health. 2024;6:e12-e22. 10.1016/S2589-7500(23)00225-X 38123252

[33] Cirillo D , Valencia A, Villegas M, et al Sex and gender bias in natural language processing. In: *Sex and Gender Bias in Technology and Artificial Intelligence: Biomedicine and Healthcare Applications*. Elsevier; 2022:113–132.

[34] Vaidya A , Chen RJ, Williamson DFK, et al Demographic bias in misdiagnosis by computational pathology models. Nat Med. 2024;30:1174-1190. 10.1038/s41591-024-02885-z 38641744

