# Supplementary material for: Development of a benchmarking dataset for symptom detection using large language models
Source: JAMIA Open. 2026 Jul 10;9(4):ooag134. doi: 10.1093/jamiaopen/ooag134 (PMC13354605; doi:10.1093/jamiaopen/ooag134)
Supplement: ooag134_Supplementary_Data [file ooag134_supplementary_data.zip › Symptoms-AI_JAMIA-Open_Supplemental-Table-1_10-6-25.docx]

**Supplemental Table 1: Codebook**

| **Symptom** | **Definition** | **Illustrative Examples** | **Other Notes & Counterexamples** |
| --- | --- | --- | --- |
| Anxiety | [CTCAE]: A disorder characterized by apprehension of danger and dread accompanied by restlessness, tension, tachycardia, and dyspnea unattached to a clearly identifiable stimulus.  Include both mentions of anxiety as a historical symptom/condition and as a current or hypothetical symptom.   Include mention of panic attacks by name (do not infer from symptoms of panic attacks if anxiety not mentioned in excerpt, as these could be attributed to other conditions out-of-context).  This is a challenging / complex symptom which is extremely context specific. Don't infer anxiety from expressions like “worry” or “afraid” unless it's unambiguously clear that anxiety is the present symptom. | *Positive Uses:*  “any past medical, any conditions, medical conditions?” / “uh, depression and anxiety”; “and have you had depression or anxiety in the past?”; “I've got acid reflux and uh, I also have anxiety”  *Example:*  “D: OK, and you can recognize those signs like feeling jittery and sweaty might, maybe a little anxious when that comes on?    P: Yes, I'm an expert now.”  Batch 1 (RES0181) | *Negative Uses:*  “I am worried about X (acute situation)”; “stress”  *Notes:*  Stress = not a symptom despite interesting/ relevant to exacerbating symptoms |
| Concentration Problems | [CTCAE] (Concentration Impairment): A disorder characterized by a deterioration in the ability to concentrate.  Include statements such as “hard time paying attention”, “trouble concentrating”, etc.  Include explicit statements; don't infer unless concentration problems are clearly discussed in the excerpt in question. | *Positive Uses:*  “any issues concentrating on tasks?”  *Example:*  “D: OK. How is your concentration been?  P: Uhm, not great.  D: OK. You haven't been able to focus and have you felt like things are moving in slow motion?”  Batch 42 (RES0174) |  |
| Constipation | [CTCAE]: A disorder characterized by irregular and infrequent or difficult evacuation of the bowels.  Include statements like “straining with bowel movements”, “hard stools”, “difficult evacuation”  Do not include general statements such as “changes in bowel habits” unless it specifies constipation. | *Positive Uses:*  “any diarrhea or constipation?”; “any changes in your bowel movements, any constipation or diarrhea?”  *Example:*  “D: Okay, and have you been sick at all recently, either with um, any cold-like symptoms uh, or any GI symptoms, like diarrhea, um, constipation, anything like that?  P: Uh, no, I haven't had any, anything like that.”  Batch 25 (MSK0043) | *Negative Uses:*  “any changes in your bowel movements?” (no other context)  *Notes:*  Mention of general “GI issues” or “blood in stool” should be coded as Other. |
| Cough | [CTCAE]: A disorder characterized by sudden, often repetitive, spasmodic contraction of the thoracic cavity, resulting in violent release of air from the lungs and usually accompanied by a distinctive sound. | *Positive Uses:*  “had you had a cough”; “no cough like now, uhm, I I did have a slight cough last week”; “I've had a cough for the past three days”  *Example:*  “D: How about a sore throat or a cough?  P: No, OK.”  Batch 15 (MSK0036) | *Notes:*  Do not include “cold” without mention of cough; mention of “sputum” or “blood in sputum” seen as a part of cough discussion should be coded as Other. |
| Diarrhea | [CTCAE]: A disorder characterized by an increase in frequency and/or loose or watery bowel movements.  Include statements like “having to go many times a day” (in reference to bowel movements rather than urination), runny/loose stools.  Do not include general statements such as “changes in bowel habits” unless it specifies diarrhea. | *Positive Uses:*  “any diarrhea or constipation?”; “any changes in your bowel movements, any constipation or diarrhea?”; “any changes in your bowel movements?” / “umm, yeah, well, I had diarrhea this morning actually”  *Example:*  “D: Constipation or diarrhea?  P: No.”  Batch 19 (RES0068) | *Negative Uses:*  “any changes in your bowel movements?” (no other context)  *Notes:*  Code mention of blood in stool as Other. |
| Fatigue | [CTCAE]: A disorder characterized by a state of generalized weakness with a pronounced inability to summon sufficient energy to accomplish daily activities.  Fatigue may be referenced via “fatigue” but also by expressions like being “very tired” and “having low energy”.   Sleeping X hours a day / sleeping more than usual may also indicate fatigue - if this is clearly in reference to sleepiness (rather than other sleep symptoms, insomnia, etc.), code as fatigue. | *Positive Uses:*  “any increased fatigue?”; “how has your energy been?”; “and so are you having, do you feel like tired throughout the day or increased fatigue?”  *Example:*  “D: I see, OK, and how has your energy been? Have you had uh significant fatigue?  P: Yes, I would say I am significantly more tired than I used to be.”  Batch 12 (RES0019) | *Notes:*  Code unclear sleep/wake symptoms as Other. |
| Fever | [CTCAE] A disorder characterized by elevation of the body’s temperature above the upper limit of normal.  High temperature / running a temperature is indicative of a fever.  Fever and chills are often mentioned together; chills mentioned alone should be coded as “Other”. Code expressions like “fever or chills” as both Fever and Other. | *Positive Uses:*  “do you have a fever?”; “any fevers or chills?”; “sounds like you um have had a fever as well”  *Example:*  “D: What brings you in today?  P: So two days ago I started to have this cough. Came out of nowhere. I haven't had a cough before and I, just today I've noticed the fever as well and and I've had a headache the last few days.”  Batch 51 (RES0172) | *Notes:*  Mention of “hot” without mention of temperature has insufficient context to code as fever (could be a hot flash, for example). |
| Headache | [CTCAE]: A disorder characterized by a sensation of marked discomfort in various parts of the head, not confined to the area of distribution of any nerve. | *Positive Uses:*  "do you have a headache"; "my head was aching"; "I had a migraine"  *Example:*  "D: OK, any headaches?  P: No."  Batch 7 (MSK0031) | *Notes:*  Wound-related head pain (that is clearly separate from headache) should be coded as Pain. |
| Nausea | [CTCAE]: A disorder characterized by a queasy sensation and/or the urge to vomit.  Include expressions of nausea/queasiness/like one might throw up.  “Upset stomach” = nausea, whereas “stomach hurts” = pain | *Positive Uses:*  “any nausea or vomiting?”; “I feel like throwing up”; “nausea or vomiting?”  *Example:*  “D: Nausea or vomiting?  P: No”  Batch 5 (RES0141) | *Notes:*  Code "stomach hurts" as Pain (unless you can tell it is nausea from the surrounding context)  Code nonspecific mention of “GI issues” as Other. |
| Numbness and tingling | [CTCAE] (Paresthesia): A disorder characterized by functional disturbances of sensory neurons resulting in abnormal cutaneous sensations of tingling, numbness, pressure, cold, and/or warmth.  Include mentions of changes of sensation (reference to numbness) such as “can't feel the bottom of my feet.” | *Positive Uses:*  “any numbness or tingling anywhere?”; “any tingling sensation or numbness in your back or lower limbs, in your legs?”  *Example:*  “D: OK. And are you having any other symptoms, like numbness or tingling in your extremities or back at all?  P: No.”  Batch 6 (MSK0024) |  |
| Pain | [CTCAE]: A disorder characterized by the sensation of marked discomfort, distress, or agony (note that the CTCAE contains over 40 different specific pain terms).  Pain includes mentions of pain, aches, and soreness. Includes pain ratings, modifiers that are clearly in reference to pain (e.g., sharp or dull).  Include ache and soreness expressions even in the context of muscle soreness from exercise.  Note that “Head pain” or “pain in my head” could also refer to a headache, unless these expressions are clearly a discussion of a wound or localized surgical site. “Pain in my head” should be double-coded as both pain and headache, whereas mention of headache alone should not be double-coded for pain.  Do not specify pain ratings, qualifiers, or descriptors via Other – just code as Pain. If a qualifier or rating appears without mention of pain, only code if it’s clear that what is being spoken about is Pain. | Positive Uses:  “any chest pain?”; “no pain inside your mouth or anything like that?”; “it has been a bit sore when eating food, but it hasn't been so painful to prevent me from eating”  Example:  “D: Okay, and what kind of pain is it? Is it like a sharp pain, stabbing pain, aching pain? How would you describe it?  P: Um, it's a, it's, it's like a, I don't know. I guess it's a sharp pain when I try to take a deep breath in.”  Batch 20 (RES0120) | *Negative Uses:*  “I have a headache” (headache has its own symptom category)  *Notes:*  Code “heartburn”, “reflux”, and “reduced strength”/“weakness” as Other. |
| Poor appetite | [CTCAE] (Anorexia): A disorder characterized by a loss of appetite.  Commonly asked about via “how has your appetite been” --> include as appetite being gauged.   Sometimes patients/family describe this as “not eating” / “only eating a few bites a day.” Have to be talking in the context of appetite specifically - if in reference to nausea or with an ambiguous context, code as Other. | *Positive Uses:*  “how has your appetite been?” / “also pretty low”; “the only I I guess other thing that that I forgot to mention is that he doesn't really like his appetite's been a little bit lower”; “I've been eating less than usual, just because I don't, I don't want to, you know, I don't like the pain”  *Example:*  “D: How about any loss of appetite?  P: No.”  Batch 28 (MSK0040) | *Negative Uses:*  “how has your weight been?”  *Notes:*  Appetite can be closely associated with nausea.  Don't look too far into it - code regardless of source of poor appetite (i.e., Pain). |
| Rash | [CTCAE contains multiple specific rashes, and then lists rash as a symptom of other conditions] Language used to describe rashes includes “small, raised pimples”, “blisters”, “pruritus”, “itchy or tender lesions”, “circumscribed and elevated skin lesion”, “skin eruptions.”  Include asking about skin changes. | *Positive Uses:*  “how about skin changes such as rashes”; “rashes?” / “no”; “no rashes”  *Example:*  “D: OK. How about um rashes?  P: Uh no rashes.”  Batch 44 (MSK0005) | *Negative Uses:*  “skin darkening” / “flushing” (code instead as Other) |
| Shortness of breath | [CTCAE] (Dyspnea): A disorder characterized by an uncomfortable sensation of difficulty breathing.  Include statements around difficulty breathing. | *Positive Uses:*  “any difficulties breathing?”; “how about any shortness of breath?”  *Example:*  “D: OK, are you short of breath at all?  P: No, I'm not short of breath. Um just, it's just the cough.” Batch 32 (RES0117) | *Negative Uses:*  “any wheezing?”, “respiratory problems”, “can't take a deep breath” (due to pain) should all be coded as Other. |
| Trouble drinking fluids | [No CTCAE definition]  This is a symptom of interest to oncology patients given the importance of fluid intake. This symptom is separate from difficulty swallowing or difficulty/pain when eating.  Only include if swallowing liquid / fluids specifically mentioned. | *Example:*  “D: I see, OK, so have you had any pain with swallowing food or liquids?  P: No.”  Batch 12 (RES0151) | *Negative Uses:*  “any issues swallowing food” (code as Other), “are you having any trouble or pain while swallowing any food?” (if not about fluids, code as Other (double-code the second example for Pain also)). |
| Vomiting | [CTCAE]: A disorder characterized by the reflexive act of ejecting the contents of the stomach through the mouth.  e.g., “vomiting”, “throwing up”  Nausea and vomiting are not the same, but can be related: feeling like throwing up would be Nausea, but only actually doing so qualifies as Vomiting. | *Positive Uses:*  “any nausea or vomiting”; “any headaches, nausea or vomiting?”  *Example:*  “D: OK and nausea or vomiting?  P: No.”  Batch 17 (RES0185) |  |
