# Supplementary material for: Development of a benchmarking dataset for symptom detection using large language models
Source: JAMIA Open. 2026 Jul 10;9(4):ooag134. doi: 10.1093/jamiaopen/ooag134 (PMC13354605; doi:10.1093/jamiaopen/ooag134)
Supplement: ooag134_Supplementary_Data [file ooag134_supplementary_data.zip › Symptoms-AI_JAMIA-Open_Supplemental-Table-2_10-6-25.docx]

**Supplemental Table 2: Prompts**

| **Task** | **Zero-Shot Prompt** | **Additional Settings** |
| --- | --- | --- |
| 1. Detection of general symptoms (present/absent) | “*are any medical symptoms mentioned in this transcript*” | Target output structure defined and enforced using Pydantic (code available on GitHub; see **Data Availability**). |
| 2. Detection of granular symptoms of interest (True/False for each symptom to be annotated) | “*For each symptom in symptom list return True if present and False if absent from the transcript. All symptoms required. Symptom list: 'anxiety', 'concentration problems', 'constipation', 'cough', 'diarrhea', 'fatigue', 'fever', 'headache', 'nausea', 'numbness and tingling', 'pain', 'poor appetite', 'rash', 'shortness of breath', 'trouble drinking fluids', 'vomiting', 'other'* ” |  |
