# Supplementary material for: Development of a benchmarking dataset for symptom detection using large language models
Source: JAMIA Open. 2026 Jul 10;9(4):ooag134. doi: 10.1093/jamiaopen/ooag134 (PMC13354605; doi:10.1093/jamiaopen/ooag134)
Supplement: ooag134_Supplementary_Data [file ooag134_supplementary_data.zip › Symptoms-AI_JAMIA-Open_Supplemental-Table-3_10-6-25.docx]

**Supplemental Table 3. Differences Between Model Performance**

| **Model 1** | **Model 2** | **McNemar χ²** | **p-value** | **Significant after Bonferroni correction?** | **Direction of difference** |
| --- | --- | --- | --- | --- | --- |
| GPT-4.1 Mini | GPT-4.1 | 104.0 | 3.67e-4 | **Yes** | GPT-4.1 > GPT-4.1 Mini |
| GPT-4.1 Mini | Llama-3.1 | 226.0 | 7.81e-44 | **Yes** | GPT-4.1 Mini > Llama-3.1 |
| GPT-4.1 Mini | Llama-3.2 | 208.0 | 7.58e-193 | **Yes** | GPT-4.1 Mini > Llama-3.2 |
| GPT-4.1 Mini | Llama-3.3 | 226.0 | 0.0355 | No | - |
| GPT-4.1 Mini | DeepSeek-V3 | 124.0 | 1.53e-7 | **Yes** | GPT-4.1 Mini > DeepSeek-V3 |
| GPT-4.1 Mini | Kimi K2 | 109.0 | 7.18e-7 | **Yes** | GPT-4.1 Mini > Kimi K2 |
| GPT-4.1 Mini | GPT-4.1 Nano | 120.0 | 5.25e-36 | **Yes** | GPT-4.1 Mini > GPT-4.1 Nano |
| GPT-4.1 Mini | GPT-4o Mini | 76.0 | 4.93-e14 | **Yes** | GPT-4.1 Mini > GPT-4o Mini |
| GPT-4.1 | Llama-3.1 | 284.0 | 1.30e-47 | **Yes** | GPT-4.1 > Llama-3.1 |
| GPT-4.1 | Llama-3.2 | 184.0 | 2.42e-215 | **Yes** | GPT-4.1 > Llama-3.2 |
| GPT-4.1 | Llama-3.3 | 276.0 | 3.51e-5 | **Yes** | GPT-4.1 > Llama-3.3 |
| GPT-4.1 | DeepSeek-V3 | 136.0 | 2.59e-14 | **Yes** | GPT-4.1 > DeepSeek-V3 |
| GPT-4.1 | Kimi K2 | 136.0 | 7.77e-13 | **Yes** | GPT-4.1 > Kimi K2 |
| GPT-4.1 | GPT-4.1 Nano | 102.0 | 5.36e-51 | **Yes** | GPT-4.1 > GPT-4.1 Nano |
| GPT-4.1 | GPT-4o Mini | 73.0 | 5.69e-25 | **Yes** | GPT-4.1 > GPT-4o Mini |
| Llama-3.1 | Llama-3.2 | 546.0 | 7.53e-62 | **Yes** | Llama-3.1 > Llama-3.2 |
| Llama-3.1 | Llama-3.3 | 12.0 | 7.03e-91 | **Yes** | Llama-3.1 < Llama-3.3 |
| Llama-3.1 | DeepSeek-V3 | 284.0 | 1.15e-24 | **Yes** | Llama-3.1 < DeepSeek-V3 |
| Llama-3.1 | Kimi K2 | 222.0 | 2.36e-30 | **Yes** | Llama-3.1 < Kimi K2 |
| Llama-3.1 | GPT-4.1 Nano | 572.0 | 8.92e-4 | **Yes** | Llama-3.1 > GPT-4.1 Nano |
| Llama-3.1 | GPT-4o Mini | 342.0 | 6.63e-19 | **Yes** | Llama-3.1 < GPT-4o Mini |
| Llama-3.2 | Llama-3.3 | 292.0 | 3.64e-159 | **Yes** | Llama 3.2 < Llama-3.3 |
| Llama-3.2 | DeepSeek-V3 | 248.0 | 7.12e-159 | **Yes** | Llama-3.2 < DeepSeek-V3 |
| Llama-3.2 | Kimi K2 | 265.0 | 4.53e-157 | **Yes** | Llama-3.2 < Kimi K2 |
| Llama-3.2 | GPT-4.1 Nano | 293.0 | 5.30e-111 | **Yes** | Llama-3.2 < GPT-4.1 Nano |
| Llama-3.2 | GPT-4o Mini | 274.0 | 6.09e-147 | **Yes** | Llama-3.2 < GPT-4o Mini |
| Llama-3.3 | DeepSeek-V3 | 243.0 | 0.0342 | No | - |
| Llama-3.3 | Kimi K2 | 201.0 | 0.0702 | No | - |
| Llama-3.3 | GPT-4.1 Nano | 347.0 | 2.93e-14 | **Yes** | Llama 3.3 > GPT-4.1 Nano |
| Llama-3.3 | GPT-4o Mini | 266.0 | 0.00233 | No | - |
| LDeepSeek-V3 | Kimi K2 | 169.0 | 0.593 | No | - |
| DeepSeek-V3 | GPT-4.1 Nano | 167.0 | 1.10e-15 | **Yes** | DeepSeek-V3 > GPT-4.1 Nano |
| DeepSeek-V3 | GPT-4o Mini | 145.0 | 0.160 | No | - |
| Kimi K2 | GPT-4.1 Nano | 203.0 | 3.32e-15 | **Yes** | Kimi K2 > GPT-4.1 Nano |
| Kimi K2 | GPT-4o Mini | 151.0 | 0.0503 | No | - |
| GPT-4.1 Nano | GPT-4o Mini | 137.0 | 5.58e-14 | **Yes** | GPT-4.1 Nano < GPT-4o Mini |

*Note:* Comparisons between different models, from pairwise tests. Corrected α for all tests = 0.00139. Model providers: GPT-4.1 (OpenAI), GPT-4.1 Mini (OpenAI), GPT-4.1 Nano (OpenAI), GPT-4o Mini (OpenAI), DeepSeek-V3 (DeepSeek), Llama-3.1 8B (Meta), Llama-3.2 3B (Meta), Llama-3.3 70B (Meta), Kimi K2 (Moonshot AI).
