# Supplementary material for: Development of a benchmarking dataset for symptom detection using large language models
Source: JAMIA Open. 2026 Jul 10;9(4):ooag134. doi: 10.1093/jamiaopen/ooag134 (PMC13354605; doi:10.1093/jamiaopen/ooag134)
Supplement: ooag134_Supplementary_Data [file ooag134_supplementary_data.zip › Symptoms-AI_JAMIA-Open_Supplemental-Table-4_10-6-25.docx]

**Supplemental Table 4. Performance for All Symptoms**

| **Symptom (n)** | **Model** | **Excerpt Level** | | | |
| --- | --- | --- | --- | --- | --- |
|  |  | **Precision** | **Recall** | **Accuracy** | **F1 Score** |
| **Pain**  **(n = 983)** | DeepSeek-V3 | 0.91 (0.89-0.93) | 0.63 (0.59-0.65) | 0.79 (0.78-0.81) | 0.74 (0.72-0.76) |
|  | GPT-4.1 | 0.90 (0.88-0.93) | 0.62 (0.59-0.65) | 0.79 (0.77-0.81) | 0.74 (0.71-0.76) |
|  | GPT-4.1 Mini | 0.92 (0.90-0.94) | 0.61 (0.58-0.64) | 0.79 (0.78-0.81) | 0.73 (0.71-0.76) |
|  | GPT-4.1 Nano | 0.94 (0.92-0.96) | 0.53 (0.50-0.56) | 0.76 (0.75-0.78) | 0.68 (0.65-0.71) |
|  | GPT-4o Mini | 0.91 (0.88-0.93) | 0.64 (0.61-0.67) | 0.80 (0.78-0.82) | 0.75 (0.72-0.77) |
|  | Llama-3.2 | 0.75 (0.72-0.78) | 0.60 (0.56-0.63) | 0.71 (0.69-0.73) | 0.66 (0.64-0.69) |
|  | Llama-3.3 | 0.84 (0.82-0.87) | 0.69 (0.66-0.72) | 0.79 (0.78-0.81) | 0.76 (0.74-0.78) |
|  | Llama-3.1 | 0.83 (0.81-0.86) | 0.67 (0.64-0.70) | 0.78 (0.76-0.80) | 0.74 (0.72-0.77) |
|  | Kimi K2 | 0.91 (0.89-0.93) | 0.62 (0.59-0.65) | 0.79 (0.77-0.81) | 0.74 (0.71-0.76) |
| **Cough**  **(n = 707)** | DeepSeek-V3 | 0.94 (0.92-0.96) | 0.82 (0.79-0.85) | 0.92 (0.91-0.93) | 0.87 (0.85-0.89) |
|  | GPT-4.1 | **0.98 (0.97-0.99)** | 0.80 (0.77-0.83) | 0.93 (0.92-0.94) | 0.88 (0.86-0.90) |
|  | GPT-4.1 Mini | **0.98 (0.97-0.99)** | 0.80 (0.77-0.83) | 0.93 (0.91-0.94) | 0.88 (0.86-0.90) |
|  | GPT-4.1 Nano | **0.95 (0.94-0.97)** | 0.81 (0.78-0.84) | 0.92 (0.91-0.93) | 0.87 (0.85-0.89) |
|  | GPT-4o Mini | **0.97 (0.95-0.98)** | 0.81 (0.78-0.84) | 0.93 (0.92-0.94) | 0.88 (0.86-0.90) |
|  | Llama-3.2 | 0.92 (0.90-0.94) | 0.78 (0.75-0.81) | 0.90 (0.89-0.92) | 0.84 (0.82-0.87) |
|  | Llama-3.3 | 0.89 (0.86-0.91) | 0.84 (0.81-0.86) | 0.91 (0.90-0.92) | 0.86 (0.84-0.88) |
|  | Llama-3.1 | 0.84 (0.81-0.86) | 0.86 (0.83-0.89) | 0.90 (0.88-0.91) | 0.85 (0.83-0.87) |
|  | Kimi K2 | **0.97 (0.96-0.98)** | 0.81 (0.78-0.84) | 0.93 (0.92-0.94) | 0.88 (0.87-0.90) |
| **Shortness of breath**  **(n = 520)** | DeepSeek-V3 | 0.92 (0.89-0.95) | 0.65 (0.61-0.70) | 0.90 (0.89-0.91) | 0.76 (0.73-0.80) |
|  | GPT-4.1 | 0.92 (0.89-0.95) | 0.63 (0.59-0.67) | 0.89 (0.88-0.91) | 0.75 (0.72-0.78) |
|  | GPT-4.1 Mini | **0.98 (0.96-0.99)** | 0.62 (0.57-0.66) | 0.90 (0.89-0.91) | 0.76 (0.72-0.79) |
|  | GPT-4.1 Nano | 0.94 (0.92-0.97) | 0.66 (0.62-0.70) | 0.90 (0.89-0.92) | 0.78 (0.74-0.81) |
|  | GPT-4o Mini | 0.93 (0.91-0.96) | 0.67 (0.63-0.71) | 0.91 (0.89-0.92) | 0.78 (0.75-0.81) |
|  | Llama-3.2 | 0.94 (0.91-0.96) | 0.61 (0.57-0.65) | 0.89 (0.88-0.91) | 0.74 (0.71-0.77) |
|  | Llama-3.3 | 0.85 (0.82-0.88) | 0.68 (0.64-0.72) | 0.89 (0.88-0.90) | 0.76 (0.73-0.79) |
|  | Llama-3.1 | 0.85 (0.82-0.89) | 0.68 (0.64-0.72) | 0.89 (0.88-0.90) | 0.75 (0.73-0.78) |
|  | Kimi K2 | 0.92 (0.89-0.95) | 0.65 (0.60-0.69) | 0.90 (0.89-0.91) | 0.76 (0.73-0.79) |
| **Fever**  **(n = 437)** | DeepSeek-V3 | 0.94 (0.92-0.97) | 0.58 (0.53-0.62) | 0.90 (0.89-0.92) | 0.71 (0.67-0.75) |
|  | GPT-4.1 | 0.93 (0.90-0.96) | 0.51 (0.46-0.56) | 0.89 (0.87-0.90) | 0.66 (0.61-0.70) |
|  | GPT-4.1 Mini | **0.97 (0.94-0.99)** | 0.49 (0.44-0.53) | 0.89 (0.87-0.90) | 0.65 (0.60-0.69) |
|  | GPT-4.1 Nano | 0.94 (0.91-0.97) | 0.50 (0.45-0.54) | 0.89 (0.87-0.90) | 0.65 (0.61-0.69) |
|  | GPT-4o Mini | **0.96 (0.94-0.98)** | 0.54 (0.49-0.58) | 0.90 (0.89-0.91) | 0.69 (0.65-0.73) |
|  | Llama-3.2 | 0.90 (0.86-0.94) | 0.49 (0.44-0.53) | 0.88 (0.87-0.89) | 0.63 (0.59-0.67) |
|  | Llama-3.3 | 0.92 (0.89-0.95) | 0.58 (0.54-0.63) | 0.90 (0.89-0.91) | 0.71 (0.68-0.75) |
|  | Llama-3.1 | 0.90 (0.87-0.93) | 0.57 (0.52-0.62) | 0.90 (0.88-0.91) | 0.70 (0.66-0.74) |
|  | Kimi K2 | 0.94 (0.90-0.96) | 0.53 (0.48-0.57) | 0.89 (0.88-0.91) | 0.68 (0.64-0.72) |
| **Headache**  **(n = 248)** | DeepSeek-V3 | **0.95 (0.91-0.98)** | 0.44 (0.38-0.50) | 0.93 (0.92-0.94) | 0.60 (0.54-0.65) |
|  | GPT-4.1 | **0.99 (0.97-1.00)** | 0.43 (0.37-0.49) | 0.93 (0.92-0.94) | 0.60 (0.54-0.66) |
|  | GPT-4.1 Mini | **0.97 (0.94-1.00)** | 0.42 (0.36-0.49) | 0.93 (0.92-0.94) | 0.59 (0.53-0.65) |
|  | GPT-4.1 Nano | **0.95 (0.91-0.98)** | 0.47 (0.41-0.53) | 0.93 (0.92-0.94) | 0.63 (0.57-0.68) |
|  | GPT-4o Mini | **0.95 (0.90-0.98)** | 0.42 (0.37-0.48) | 0.93 (0.92-0.94) | 0.58 (0.53-0.64) |
|  | Llama-3.2 | 0.31 (0.26-0.35) | 0.48 (0.42-0.54) | 0.81 (0.79-0.83) | 0.37 (0.33-0.42) |
|  | Llama-3.3 | 0.85 (0.78-0.91) | 0.44 (0.38-0.50) | 0.92 (0.91-0.93) | 0.58 (0.52-0.64) |
|  | Llama-3.1 | 0.71 (0.64-0.77) | 0.50 (0.44-0.56) | 0.92 (0.90-0.93) | 0.58 (0.53-0.64) |
|  | Kimi K2 | **0.97 (0.94-1.00)** | 0.43 (0.37-0.49) | 0.93 (0.92-0.94) | 0.59 (0.53-0.65) |
| **Vomiting**  **(n = 225)** | DeepSeek-V3 | 0.82 (0.67-0.96) | 0.10 (0.07-0.14) | 0.90 (0.89-0.91) | 0.18 (0.12-0.24) |
|  | GPT-4.1 | **1.00 (1.00-1.00)** | 0.10 (0.07-0.14) | 0.90 (0.89-0.91) | 0.19 (0.13-0.25) |
|  | GPT-4.1 Mini | **1.00 (1.00-1.00)** | 0.10 (0.07-0.14) | 0.90 (0.89-0.91) | 0.19 (0.13-0.25) |
|  | GPT-4.1 Nano | **1.00 (1.00-1.00)** | 0.14 (0.09-0.18) | 0.91 (0.90-0.92) | 0.24 (0.17-0.31) |
|  | GPT-4o Mini | **1.00 (1.00-1.00)** | 0.11 (0.07-0.15) | 0.90 (0.89-0.92) | 0.19 (0.13-0.26) |
|  | Llama-3.2 | 0.70 (0.56-0.84) | 0.12 (0.08-0.16) | 0.90 (0.89-0.91) | 0.20 (0.14-0.26) |
|  | Llama-3.3 | **0.96 (0.87-1.00)** | 0.11 (0.07-0.15) | 0.90 (0.89-0.92) | 0.19 (0.13-0.26) |
|  | Llama-3.1 | 0.68 (0.57-0.79) | 0.21 (0.16-0.26) | 0.90 (0.89-0.92) | 0.32 (0.25-0.39) |
|  | Kimi K2 | **1.00 (1.00-1.00)** | 0.11 (0.07-0.15) | 0.90 (0.89-0.92) | 0.19 (0.13-0.26) |
| **Nausea**  **(n = 219)** | DeepSeek-V3 | **1.00 (1.00-1.00)** | 0.14 (0.09-0.18) | 0.91 (0.90-0.92) | 0.24 (0.17-0.31) |
|  | GPT-4.1 | **1.00 (1.00-1.00)** | 0.14 (0.09-0.18) | 0.91 (0.90-0.92) | 0.24 (0.17-0.31) |
|  | GPT-4.1 Mini | **1.00 (1.00-1.00)** | 0.13 (0.09-0.18) | 0.91 (0.90-0.92) | 0.23 (0.17-0.30) |
|  | GPT-4.1 Nano | 0.93 (0.85-0.98) | 0.23 (0.18-0.29) | 0.92 (0.91-0.93) | 0.37 (0.30-0.44) |
|  | GPT-4o Mini | 0.92 (0.83-1.00) | 0.16 (0.12-0.21) | 0.91 (0.90-0.92) | 0.27 (0.20-0.34) |
|  | Llama-3.2 | 0.36 (0.27-0.45) | 0.17 (0.13-0.22) | 0.88 (0.87-0.89) | 0.23 (0.17-0.29) |
|  | Llama-3.3 | 0.94 (0.85-1.00) | 0.15 (0.10-0.19) | 0.91 (0.90-0.92) | 0.25 (0.18-0.32) |
|  | Llama-3.1 | 0.67 (0.57-0.77) | 0.24 (0.19-0.30) | 0.91 (0.90-0.92) | 0.36 (0.29-0.43) |
|  | Kimi K2 | **0.97 (0.89-1.00)** | 0.14 (0.10-0.19) | 0.91 (0.90-0.92) | 0.25 (0.18-0.32) |
| **Fatigue**  **(n = 215)** | DeepSeek-V3 | 0.86 (0.81-0.91) | 0.76 (0.71-0.82) | **0.96 (0.95-0.97)** | 0.81 (0.77-0.85) |
|  | GPT-4.1 | 0.74 (0.68-0.80) | 0.75 (0.68-0.80) | **0.95 (0.94-0.96)** | 0.74 (0.70-0.79) |
|  | GPT-4.1 Mini | 0.88 (0.83-0.93) | 0.73 (0.67-0.79) | **0.96 (0.95-0.97)** | 0.80 (0.75-0.84) |
|  | GPT-4.1 Nano | 0.82 (0.77-0.87) | 0.81 (0.75-0.85) | **0.96 (0.95-0.97)** | 0.81 (0.77-0.85) |
|  | GPT-4o Mini | 0.72 (0.66-0.78) | 0.80 (0.74-0.85) | **0.95 (0.94-0.96)** | 0.76 (0.71-0.80) |
|  | Llama-3.2 | 0.28 (0.24-0.31) | 0.81 (0.75-0.86) | 0.76 (0.75-0.78) | 0.41 (0.37-0.45) |
|  | Llama-3.3 | 0.63 (0.57-0.69) | 0.81 (0.75-0.86) | 0.93 (0.92-0.94) | 0.71 (0.66-0.75) |
|  | Llama-3.1 | 0.51 (0.46-0.56) | 0.87 (0.83-0.92) | 0.90 (0.89-0.91) | 0.65 (0.60-0.69) |
|  | Kimi K2 | 0.86 (0.81-0.90) | 0.76 (0.70-0.81) | **0.96 (0.95-0.97)** | 0.81 (0.76-0.85) |
| **Rash**  **(n = 198)** | DeepSeek-V3 | 0.93 (0.83-1.00) | 0.19 (0.14-0.24) | 0.92 (0.91-0.93) | 0.31 (0.24-0.39) |
|  | GPT-4.1 | 0.88 (0.76-0.97) | 0.18 (0.12-0.23) | 0.92 (0.91-0.93) | 0.30 (0.21-0.37) |
|  | GPT-4.1 Mini | 0.82 (0.69-0.92) | 0.18 (0.12-0.23) | 0.92 (0.91-0.93) | 0.29 (0.21-0.37) |
|  | GPT-4.1 Nano | 0.81 (0.69-0.91) | 0.23 (0.18-0.30) | 0.92 (0.91-0.93) | 0.36 (0.28-0.44) |
|  | GPT-4o Mini | 0.76 (0.63-0.87) | 0.21 (0.15-0.27) | 0.92 (0.91-0.93) | 0.33 (0.25-0.40) |
|  | Llama-3.2 | 0.93 (0.84-1.00) | 0.19 (0.14-0.25) | 0.92 (0.91-0.93) | 0.32 (0.24-0.39) |
|  | Llama-3.3 | 0.83 (0.72-0.93) | 0.22 (0.17-0.28) | 0.92 (0.91-0.93) | 0.35 (0.28-0.42) |
|  | Llama-3.1 | 0.71 (0.59-0.81) | 0.25 (0.19-0.31) | 0.92 (0.91-0.93) | 0.37 (0.29-0.44) |
|  | Kimi K2 | 0.90 (0.79-0.98) | 0.18 (0.13-0.24) | 0.92 (0.91-0.93) | 0.30 (0.23-0.38) |
| **Diarrhea**  **(n = 182)** | DeepSeek-V3 | **0.97 (0.90-1.00)** | 0.18 (0.12-0.23) | 0.93 (0.92-0.94) | 0.30 (0.22-0.38) |
|  | GPT-4.1 | 0.94 (0.84-1.00) | 0.17 (0.12-0.23) | 0.93 (0.91-0.94) | 0.29 (0.21-0.37) |
|  | GPT-4.1 Mini | 0.94 (0.84-1.00) | 0.17 (0.12-0.23) | 0.93 (0.91-0.94) | 0.29 (0.21-0.37) |
|  | GPT-4.1 Nano | 0.94 (0.85-1.00) | 0.18 (0.12-0.24) | 0.93 (0.92-0.94) | 0.30 (0.22-0.38) |
|  | GPT-4o Mini | **0.97 (0.90-1.00)** | 0.18 (0.12-0.23) | 0.93 (0.92-0.94) | 0.30 (0.22-0.38) |
|  | Llama-3.2 | 0.90 (0.78-0.98) | 0.19 (0.13-0.24) | 0.93 (0.92-0.94) | 0.31 (0.23-0.38) |
|  | Llama-3.3 | 0.84 (0.72-0.95) | 0.18 (0.12-0.23) | 0.93 (0.91-0.94) | 0.29 (0.21-0.37) |
|  | Llama-3.1 | 0.84 (0.75-0.94) | 0.24 (0.18-0.31) | 0.93 (0.92-0.94) | 0.37 (0.29-0.46) |
|  | Kimi K2 | 0.89 (0.77-0.97) | 0.18 (0.12-0.23) | 0.93 (0.91-0.94) | 0.29 (0.22-0.37) |
| **Poor appetite**  **(n = 138)** | DeepSeek-V3 | 0.83 (0.74-0.91) | 0.45 (0.37-0.53) | **0.96 (0.95-0.97)** | 0.58 (0.50-0.66) |
|  | GPT-4.1 | 0.80 (0.72-0.89) | 0.44 (0.36-0.52) | **0.96 (0.95-0.96)** | 0.57 (0.49-0.64) |
|  | GPT-4.1 Mini | 0.83 (0.74-0.91) | 0.45 (0.37-0.53) | **0.96 (0.95-0.97)** | 0.58 (0.50-0.66) |
|  | GPT-4.1 Nano | 0.81 (0.72-0.90) | 0.40 (0.32-0.48) | **0.95 (0.95-0.96)** | 0.53 (0.45-0.61) |
|  | GPT-4o Mini | 0.76 (0.67-0.85) | 0.46 (0.39-0.54) | **0.95 (0.95-0.96)** | 0.58 (0.50-0.65) |
|  | Llama-3.2 | 0.32 (0.26-0.38) | 0.51 (0.43-0.59) | 0.89 (0.88-0.91) | 0.39 (0.33-0.46) |
|  | Llama-3.3 | 0.77 (0.67-0.85) | 0.49 (0.41-0.58) | **0.96 (0.95-0.97)** | 0.60 (0.52-0.68) |
|  | Llama-3.1 | 0.66 (0.58-0.74) | 0.69 (0.62-0.76) | **0.96 (0.95-0.97)** | 0.68 (0.61-0.74) |
|  | Kimi K2 | 0.85 (0.77-0.93) | 0.45 (0.37-0.53) | **0.96 (0.95-0.97)** | 0.59 (0.51-0.66) |
| **Constipation**  **(n = 116)** | DeepSeek-V3 |  | 0.03 (0.01-0.07) | **0.95 (0.94-0.95)** |  |
|  | GPT-4.1 |  | 0.03 (0.01-0.07) | **0.95 (0.94-0.96)** |  |
|  | GPT-4.1 Mini |  | 0.03 (0.01-0.07) | **0.95 (0.94-0.96)** |  |
|  | GPT-4.1 Nano | 0.60 (0.27-0.91) | 0.05 (0.02-0.09) | **0.95 (0.93-0.95)** |  |
|  | GPT-4o Mini |  | 0.03 (0.01-0.07) | **0.95 (0.94-0.95)** |  |
|  | Llama-3.2 | 0.88 (0.60-1.00) | 0.06 (0.02-0.11) | **0.95 (0.94-0.96)** |  |
|  | Llama-3.3 | 0.44 (0.11-0.80) | 0.03 (0.01-0.07) | 0.94 (0.93-0.95) |  |
|  | Llama-3.1 | 0.75 (0.55-0.93) | 0.13 (0.07-0.19) | **0.95 (0.94-0.96)** | 0.22 (0.13-0.31) |
|  | Kimi K2 |  | 0.03 (0.01-0.07) | **0.95 (0.94-0.95)** |  |
| **Numbness and tingling**  **(n = 93)** | DeepSeek-V3 | 0.83 (0.70-0.94) | 0.37 (0.27-0.47) | **0.97 (0.96-0.98)** | 0.50 (0.40-0.61) |
|  | GPT-4.1 | 0.83 (0.69-0.94) | 0.31 (0.22-0.41) | **0.97 (0.96-0.97)** | 0.45 (0.34-0.55) |
|  | GPT-4.1 Mini | 0.79 (0.66-0.91) | 0.33 (0.24-0.43) | **0.97 (0.96-0.97)** | 0.47 (0.36-0.57) |
|  | GPT-4.1 Nano | 0.74 (0.63-0.86) | 0.44 (0.34-0.54) | **0.97 (0.96-0.98)** | 0.55 (0.45-0.64) |
|  | GPT-4o Mini | 0.79 (0.65-0.92) | 0.33 (0.24-0.43) | **0.97 (0.96-0.97)** | 0.47 (0.36-0.57) |
|  | Llama-3.2 | 0.80 (0.66-0.92) | 0.31 (0.22-0.41) | **0.97 (0.96-0.97)** | 0.45 (0.33-0.56) |
|  | Llama-3.3 | 0.78 (0.65-0.89) | 0.38 (0.28-0.48) | **0.97 (0.96-0.98)** | 0.50 (0.40-0.60) |
|  | Llama-3.1 | 0.60 (0.48-0.72) | 0.45 (0.35-0.56) | **0.96 (0.95-0.97)** | 0.51 (0.41-0.61) |
|  | Kimi K2 | 0.80 (0.67-0.92) | 0.31 (0.21-0.41) | **0.97 (0.96-0.97)** | 0.45 (0.34-0.55) |
| **Trouble drinking fluids**  **(n = 27)** | DeepSeek-V3 | 0.32 (0.08-0.56) | 0.19 (0.05-0.35) | **0.98 (0.98-0.99)** |  |
|  | GPT-4.1 | 0.36 (0.09-0.62) | 0.19 (0.05-0.35) | **0.99 (0.98-0.99)** |  |
|  | GPT-4.1 Mini | 0.18 (0.00-0.43) | 0.07 (0.00-0.18) | **0.98 (0.98-0.99)** |  |
|  | GPT-4.1 Nano | 0.27 (0.08-0.46) | 0.22 (0.07-0.39) | **0.98 (0.98-0.99)** |  |
|  | GPT-4o Mini | 0.37 (0.15-0.59) | 0.26 (0.10-0.43) | **0.98 (0.98-0.99)** |  |
|  | Llama-3.2 | 0.07 (0.02-0.13) | 0.18 (0.05-0.33) | **0.96 (0.95-0.97)** |  |
|  | Llama-3.3 | 0.29 (0.10-0.50) | 0.22 (0.07-0.39) | **0.98 (0.98-0.99)** |  |
|  | Llama-3.1 | 0.33 (0.13-0.52) | 0.29 (0.12-0.47) | **0.98 (0.98-0.99)** | 0.31 (0.14-0.46) |
|  | Kimi K2 | 0.30 (0.08-0.53) | 0.19 (0.05-0.35) | **0.98 (0.98-0.99)** |  |
| **Anxiety**  **(n = 15)** | DeepSeek-V3 | 0.17 (0.07-0.28) | 0.60 (0.36-0.86) | **0.98 (0.97-0.98)** | 0.26 (0.12-0.41) |
|  | GPT-4.1 | 0.20 (0.10-0.33) | 0.67 (0.42-0.91) | **0.98 (0.97-0.98)** | 0.31 (0.16-0.46) |
|  | GPT-4.1 Mini | 0.24 (0.12-0.37) | 0.67 (0.42-0.91) | **0.98 (0.98-0.99)** | 0.35 (0.19-0.51) |
|  | GPT-4.1 Nano | 0.25 (0.12-0.40) | 0.67 (0.42-0.91) | **0.98 (0.98-0.99)** | 0.36 (0.20-0.52) |
|  | GPT-4o Mini | 0.20 (0.10-0.32) | 0.74 (0.50-0.94) | **0.98 (0.97-0.98)** | 0.31 (0.17-0.45) |
|  | Llama-3.2 | 0.57 (0.29-0.85) | 0.54 (0.29-0.80) | **0.99 (0.99-1.00)** |  |
|  | Llama-3.3 | 0.36 (0.21-0.54) | 0.80 (0.58-1.00) | **0.99 (0.98-0.99)** | 0.50 (0.32-0.66) |
|  | Llama-3.1 | 0.38 (0.21-0.57) | 0.74 (0.50-0.94) | **0.99 (0.98-0.99)** | 0.50 (0.31-0.67) |
|  | Kimi K2 | 0.25 (0.13-0.38) | 0.74 (0.50-0.94) | **0.98 (0.98-0.99)** | 0.36 (0.21-0.52) |
| **Concentration problems**  **(n = 6)** | DeepSeek-V3 | 0.67 (0.33-1.00) |  | **1.00 (1.00-1.00)** |  |
|  | GPT-4.1 |  |  | **1.00 (1.00-1.00)** |  |
|  | GPT-4.1 Mini | 0.45 (0.14-0.78) |  | **1.00 (0.99-1.00)** |  |
|  | GPT-4.1 Nano | 0.37 (0.14-0.62) |  | **1.00 (0.99-1.00)** |  |
|  | GPT-4o Mini | 0.43 (0.17-0.69) |  | **1.00 (0.99-1.00)** |  |
|  | Llama-3.2 | 0.55 (0.20-0.88) |  | **1.00 (1.00-1.00)** |  |
|  | Llama-3.3 | 0.50 (0.20-0.80) |  | **1.00 (0.99-1.00)** |  |
|  | Llama-3.1 | 0.38 (0.14-0.65) |  | **1.00 (0.99-1.00)** |  |
|  | Kimi K2 |  |  | **1.00 (1.00-1.00)** |  |

*Note:* Values of 0.95 and higher are highlighted in bold, with an asterisk on bolded values from rarer symptoms (few samples). Blank cells crossed out diagonally indicate too few values captured during bootstrapping to calculate metric. Models and providers: GPT-4.1 (OpenAI), GPT-4.1 Mini (OpenAI), GPT-4.1 Nano (OpenAI), GPT-4o Mini (OpenAI), DeepSeek-V3 (DeepSeek), Llama-3.1 8B (Meta), Llama-3.2 3B (Meta), Llama-3.3 70B (Meta), Kimi K2 (Moonshot AI).
