# Supplementary material for: Development of a benchmarking dataset for symptom detection using large language models
Source: JAMIA Open. 2026 Jul 10;9(4):ooag134. doi: 10.1093/jamiaopen/ooag134 (PMC13354605; doi:10.1093/jamiaopen/ooag134)
Supplement: ooag134_Supplementary_Data [file ooag134_supplementary_data.zip › Symptoms-AI_JAMIA-Open_Supplemental-Table-5_10-6-25.docx]

**Supplemental Table 5. Manual Error Review for GPT-4.1 Outputs**

|  | **Notes from Manual Error Review (GPT-4.1)** | |
| --- | --- | --- |
| **Specific Symptoms** | **False Positives (n=10 per symptom)** | **False Negatives (n=10 per symptom)** |
| Pain | Mention of medication without mention of the symptom itself; tough cases requiring annotator discussions/ codebook decisions; symptom implied but not explicitly mentioned; ambiguous or potentially relevant mention excluded by annotators (e.g., sore, sore throat, ache). | Symptom denied by patient (e.g., ‘do you have chest pain?’/‘no’), mentioned as hypothetically occurring in the future (e.g., ‘if you have a cough, be sure to call’), or with an ambiguous status (i.e., asked about at the very end of the excerpt, with no answer); symptoms explicitly mentioned in long list of symptom questions (no real pattern seen in regard to where in the transcript errors tended to be – some were at the beginning, some at the end, some in the middle). No false negatives were explicitly mentioned symptoms confirmed by the patient as present. |
|  |  |  |
|  |  |  |
|  |  |  |
| Cough | Mention of medication without mention of the symptom itself; ambiguous or potentially relevant mention excluded by annotators (e.g., phlegm/sputum, coughing up blood, cold); reference to person besides the patient with the symptom (i.e., doctor asking about potential recent exposures in the context of tuberculosis risk); awkward mention of symptom in distant past (not relevant as an encounter symptom). |  |
|  |  |  |
|  |  |  |
|  |  |  |
| Shortness of breath | Potentially relevant mentions excluded by annotators (e.g., wheeze) or deemed insufficient alone (e.g., breathing faster, trying to breathe); cases where shortness of breath potentially related but not explicitly mentioned, insufficiently implied (e.g., pain/catching breath, chest tightness, allergic reaction/throat swelling); discussion of conditions that involve or imply shortness of breath (e.g., COPD, asthma; mention of ventolin). |  |
|  |  |  |
|  |  |  |
|  |  |  |

*Note:* In this table, observations of manual error review for GPT-4.1 errors are shown. Most false positives were difficult cases that were clarified by annotator use of a codebook, discussions, and a third-party adjudication process for disagreements; most false negatives referenced a denied or ambiguous symptom. Both types of errors would likely be reduced through prompt engineering & optimization.
